# Functional annotation of the transcriptome of *Sorghum bicolor *in response to osmotic stress and abscisic acid

**DOI:** 10.1186/1471-2164-12-514

**Published:** 2011-10-18

**Authors:** Diana V Dugas, Marcela K Monaco, Andrew Olsen, Robert R Klein, Sunita Kumari, Doreen Ware, Patricia E Klein

**Affiliations:** 1 Department of Horticulture, Texas A&M University, College Station, TX 77843, USA; 2 Cold Spring Harbor Laboratory, Cold Spring Harbor, NY 11724, USA; 3 USDA-ARS, Southern Plains Agricultural Research Center, College Station, TX 77843, USA; 4 USDA-ARS, Robert W. Holley Center for Agriculture and Health, Cornell University, Ithaca, NY 14853, USA

## Abstract

**Background:**

Higher plants exhibit remarkable phenotypic plasticity allowing them to adapt to an extensive range of environmental conditions. Sorghum is a cereal crop that exhibits exceptional tolerance to adverse conditions, in particular, water-limiting environments. This study utilized next generation sequencing (NGS) technology to examine the transcriptome of sorghum plants challenged with osmotic stress and exogenous abscisic acid (ABA) in order to elucidate genes and gene networks that contribute to sorghum's tolerance to water-limiting environments with a long-term aim of developing strategies to improve plant productivity under drought.

**Results:**

RNA-Seq results revealed transcriptional activity of 28,335 unique genes from sorghum root and shoot tissues subjected to polyethylene glycol (PEG)-induced osmotic stress or exogenous ABA. Differential gene expression analyses in response to osmotic stress and ABA revealed a strong interplay among various metabolic pathways including abscisic acid and 13-lipoxygenase, salicylic acid, jasmonic acid, and plant defense pathways. Transcription factor analysis indicated that groups of genes may be co-regulated by similar regulatory sequences to which the expressed transcription factors bind. We successfully exploited the data presented here in conjunction with published transcriptome analyses for rice, maize, and Arabidopsis to discover more than 50 differentially expressed, drought-responsive gene orthologs for which no function had been previously ascribed.

**Conclusions:**

The present study provides an initial assemblage of sorghum genes and gene networks regulated by osmotic stress and hormonal treatment. We are providing an RNA-Seq data set and an initial collection of transcription factors, which offer a preliminary look into the cascade of global gene expression patterns that arise in a drought tolerant crop subjected to abiotic stress. These resources will allow scientists to query gene expression and functional annotation in response to drought.

## Background

Crop productivity is significantly impacted by abiotic constraints, especially water availability [1]. Given the expanding demand for water by urban populations [2,3], crop productivity in drought-prone environments must be addressed primarily through genetic improvement [1]. The genetic basis of plant adaptation to the environment is complex and includes an extraordinary range of developmental strategies (i.e., cacti, ephemerals, lichens), morphological features (i.e., variation in leaf and root system morphology), biochemical mechanisms (i.e., C3/C4/CAM photosynthesis, osmotic adjustment, dehydrins), and physiological traits (i.e., stomatal regulation, stay-green) (reviewed in [1,4-11]).

Sorghum (*Sorghum bicolor *L. Moench) is an excellent model for the study of plant response to abiotic stress, particularly drought stress. With the exception of millet, sorghum is the cereal best adapted to water-limited environments and ranks amongst the most drought tolerant of all crops grown in the U.S. [9]. Sorghum's drought tolerance is consistent with its evolution in Africa [12], which resulted in the development of heritable morphological and anatomical characteristics (i.e., C4 photosynthesis, thick leaf wax, deep root system) that permit sorghum's growth in hot, dry environments. Sorghum also exhibits physiological responses (i.e., osmotic adjustment, stay-green) that allow continued growth under drought, and adaptive mechanisms (i.e., quiescence) that allow extreme drought tolerance.

A challenge presently facing plant scientists is to obtain the molecular knowledge and experimental tools required to identify the network of genes that condition crop adaptation to harsh environments. The interplay between drought and changes in plant gene expression has been intensely studied in numerous species including Arabidopsis [13-22], rice [23-27], maize [28-30], and sorghum [31]. Recently, the metabolic interplay between ABA and other plant hormones was implicated in a variety of plants grown under drought or osmotic stress conditions [32-34]. These studies provide insight into the relationship among drought tolerance, gene networks, and the metabolic pathways conditioning each species response to drought (reviewed in [35-39]).

In the mid-2000's, cDNA microarray experiments were conducted to examine sorghum's response to various abiotic and biotic stresses. These studies included the effects of PEG-induced osmotic stress, exogenous abscisic acid (ABA), salt, wounding (by jasmonic acid - JA), and insects (by salicylic acid - SA) [31,40-42]. The recent release of a complete genome sequence for sorghum [43] and the development of SorghumCyc, a metabolic pathways database (http://www.gramene.org/pathway/sorghumcyc.html), as well as ultra high-throughput sequencing technology (i.e., next generation sequencing or NGS) provide a unique opportunity to obtain a more complete view of the genes and gene networks conditioning abiotic stress tolerance in sorghum.

Herein, we exploited RNA-Seq technology in combination with the sorghum genome sequence [43] and the SorghumCyc metabolic pathways database to characterize the sorghum transcriptome and to reexamine the differential expression of sorghum genes in response to exogenous ABA and osmotic stress. The present results expand on the sorghum cDNA-array analyses of Buchanan et al. [31] by examining the expression of all currently annotated sorghum genes, providing evidence of the interconnectivity of drought-regulated pathways, and discussing the interplay between transcription factors (TFs) and the corresponding *cis*-acting elements upon which they act. We also employ the sequenced genomes of sorghum, rice, maize, and Arabidopsis to explore orthologous transcripts from genes that exhibit differential expression following ABA treatment and/or osmotic stress across species to investigate the possible evolutionary significance of genes of unknown function in abiotic stress response.

## Results and Discussion

### Mapping the *Sorghum bicolor *Transcriptome

In 2005, changes in sorghum gene expression due to exogenous ABA treatment and PEG-induced osmotic stress were assayed by cDNA microarray technology [31], and, based on existing EST resources at that time, it was estimated that the sorghum cDNA array consisted of 12,982 unique genes [31]. In 2009, the sorghum draft genome sequence and annotations revealed ~34,500 genes [43]; upon reexamination of cDNA sequences on the sorghum microarray, we determined that only ~25% of the reported sorghum genes (8,797 EST-to-unique gene mappings) were spotted on the cDNA array. Due to the limited number of unique genes spotted on the sorghum cDNA microarray, the present study readdresses the changes in sorghum gene expression in response to exogenous ABA or osmotic stress using a global transcriptome profiling approach.

**Figure 1:**
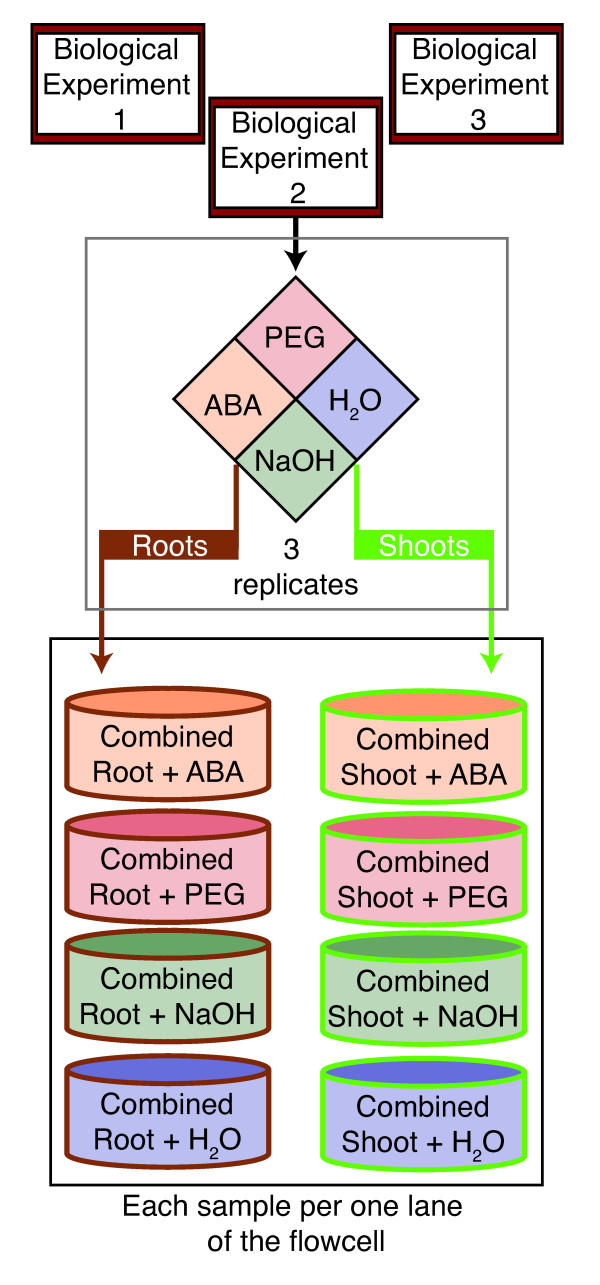
**Experimental design and replication**. The experiments were conducted three times (top three red-outlined boxes). For each experiment, three hydroponic buckets were treated with control (NaOH and H_2_O) or treatment (ABA and PEG) (middle grey box; colored diamond). After 27 hrs of treatment, 10 plants from each bucket were harvested, separated into roots and shoots, and RNA extracted (middle grey box; brown and green arrows). RNA samples from each bucket were combined in equimolar amounts and 5 μg of combined RNA used to create the Illumina RNA-Seq cDNA (bottom box; colored bins). Each flowcell (8 lanes) contained 4 root and 4 shoot samples, each having been treated with ABA, NaOH, PEG, or H_2_O for 27 hrs. The order of the samples on the flowcell was assigned by random draw for each Illumina run.

**Figure 2:**
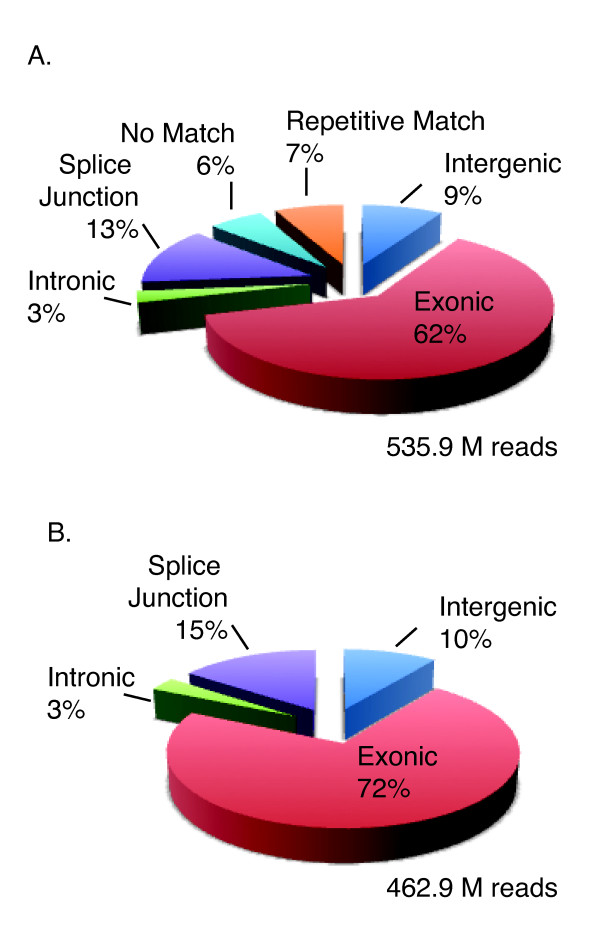
**RNA-Seq analysis of the *Sorghum bicolor *transcriptome**. Distribution of the total number of all sequencing reads that passed Illumina's filtering among annotated features across the sorghum genome (A). Distribution of the total number of all sequencing reads that passed Illumina's filtering and aligned uniquely to the sorghum genome (B).

We conducted RNA-Seq on three independent biological experiments, each one consisting of a pool of paired shoot/root tissues treated with ABA, PEG or their respective controls (Figure 1). A total of 689.5 million reads were generated across all three biological replicates. These sequences were trimmed to 50bp and aligned to the sorghum genome. Of the total reads, 535.9 million passed purity filtering standards, and, of those, 462.9 million (~86-87%) uniquely mapped to the sorghum genome (Figure 2A, Table 1 and Additional File 1). Most of the uniquely mapped RNA-Seq reads aligned to exons (72%) with the remainder distributed among introns (3%), intergenic regions (10%), and splice junctions (15%) (Figure 2B and Additional File 1). In total, 67.1% of the 689.5 million collected reads passed filtering and mapped uniquely to the genome, slightly higher than previously published results, which range from 38-60% [44-47].

**Table 1:** Summary of the RNA-Seq Data Combined from Three Independent Biological Experiments

	NaOH-treated		ABA-treated		H_2_O-treated		PEG-treated	
	
	Shoot	Root	Shoot	Root	Shoot	Root	Shoot	Root
Total 50bp single end reads (M)	87.9	90.2	80.5	84.2	88.7	88.8	87	82.2
Total reads passing Illumina's purity filtering (PF) (M)	70.7	68	64.5	65.3	66.3	67.7	67.2	66.2
Total PF reads that were "repeat masked" (M)	5.4	4.9	4.6	5.0	4.9	4.7	5.3	4.8
Total PF reads with no match to the *Sb *genome (M)	4.4	4.4	2.3	3.5	6.3	6.6	1.2	2.4
Total reads uniquely mapped to the *Sb *genome (M)	60.7	58.6	57.5	56.5	54.7	56.3	59.7	58.9
Total reads mapped to annotated genes (M)	55.59	51.78	52.31	50.32	49.59	49.74	53.89	52.38

**Figure 3:**
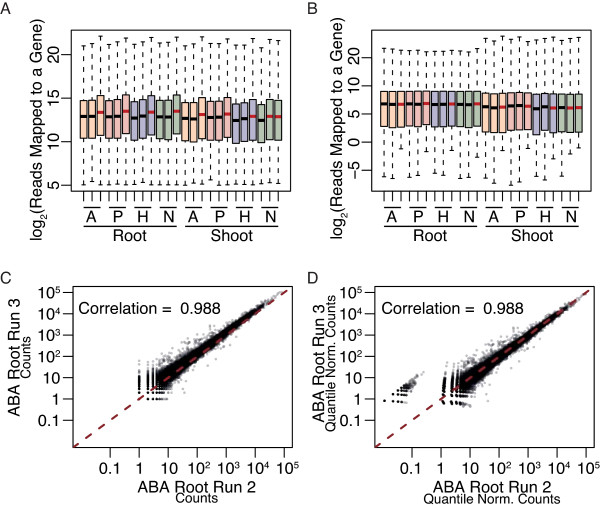
**Quantile Normalization of RNA-Seq Reads**. Box-and-whisker plots show median reads per gene (black and red bars) and varying ranges (colored boxes) for the lanes before normalization (A), which are removed after normalization (B). Red bars denote the lanes analyzed using updated RTA V1.6 software and therefore display an increase in total read counts per lane. Whiskers denote the lowest datum still within 1.5 interquartile range (IQR) of the lower quartile, and the highest datum still within 1.5 IQR of the upper quartile. Scatterplots of counts/gene between runs 2 and 3 in ABA-treated roots before (C) and after quantile normalization (D). A = ABA-treatment; P = PEG-treatment; H = H_2_O control; N = NaOH control.

An upgrade in the software used for base calling after completion of the second biological replicate resulted in an increase in the overall number of reads collected, emphasizing the need for normalization across runs. Quantile normalization was performed for each run and subsequently across all samples simultaneously (Figure 3) using edgeR [48-50]. The range of reads mapped to a gene was comparable across runs, with the 9 lanes of increased sequence counts displaying a slight increase in median read number per gene (Figure 3A and 3B, samples containing red bar). The Spearman coefficient of correlation between biological runs was high, supporting the reproducibility of the results (Figure 3C and 3D and Additional File 2). Previously published technical replicate correlation coefficients range between 0.92 and 0.96 [51-53], centered in the range that we observed between biological replicates (Spearman coefficient of correlation of 0.91 - 0.99; Additional File 2).

As reported by Paterson et al. [43], the annotation of the sorghum genome identified 34,496 gene models, with ~27,640 of these considered bona fide, or high confidence, protein-coding genes following homology-based and *ab initio *gene prediction methods combined with EST support from various cereals. The remaining ~6,850 sorghum gene models predicted from the genome sequence are considered low confidence [43] due to a lack of any additional support. In the present study, we were able to resolve 34,144 of the 34,496 gene models as a result of the lack of strand-specific information in the RNA-Seq cDNA. Unless specifically stated, this group of genes is what we refer to as "all annotated genes". In previous studies examining the rice [45] and soybean [46] transcriptomes via RNA-Seq, transcriptionally active genes were defined as those genes with at least two uniquely mapped raw counts. When this same definition was applied in the present study, we found transcriptional activity for 28,335 unique genes when considering all samples in all runs; 25,568 high confidence and 2,649 low confidence protein-coding genes, as well as 118 non-coding pri-microRNAs [43] (see Additional File 1). Thus ~92.5% of high confidence annotated genes and ~83% of all annotated genes reported by Paterson et al. [43] showed transcriptional activity in the plant tissues and treatments examined herein. By comparison, deep sequencing resulted in verified expression of 79% and 74.2% of the annotated genes in the combined 4 maize leaf developmental zones [54] and 14 soybean tissues [46], respectively.

Of the 34,144 sorghum gene models, 5,809 (~17%) did not have any detectable transcriptional activity across all samples indicating that either these models are not expressed in any of the developmental stages/tissues examined in the present study or the models do not represent bona fide genes. Of these genes, 3,915 (~67%) are of low confidence, and 1,819 (~31%) are located in pericentromeric regions, suggesting they may be transcriptionally silent. Additional transcriptome profiling across different developmental stages/tissues (e.g., apical and vegetative meristems, developing inflorescence) and/or different abiotic/biotic environmental variables will be required to further assess the transcriptional activity of these genes.

### Differential Gene Expression Determination and Validation

To determine the appropriate read depth criteria for differential gene expression, we examined the data for trends (see Methods for further details), and a 2X median read depth cutoff in one of the two samples being compared (i.e., ABA-treated vs. NaOH-treated) was chosen for examination of differential gene expression. This cutoff value minimizes the rate of false positives while retaining genes of lower expression. In addition, only genes having a log_2_-fold change ≥ 1.0 or ≤ -1.0 and an adjusted p-value < 0.05, as determined by edgeR, were included in our analysis of differential gene expression. These restrictions yielded differentially expressed (DE) gene lists ranging from ~1,000 to nearly 3,200 genes, depending on the tissue/treatment combination (Additional File 3). Among all tissues and treatments examined, 5,156 unique genes were classified as DE (Additional File 4); including 5,018 high confidence and 123 low confidence protein-coding genes, and 15 models annotated as pri-microRNAs [43]. Approximately a third of the DE gene products (1,939 out of 5,156) are currently annotated as either predicted protein, similar to expressed protein or putative uncharacterized protein (Additional File 4) [43], which reflects the need for further proteomics studies in sorghum.

To validate the differential expression data, we performed quantitative reverse transcription PCR (qPCR) on randomly chosen mRNAs that were differentially expressed in response to ABA or osmotic stress. We conducted a total of 268 qPCR tests on a set of 157 DE genes (Additional File 5). Overall, we found a strong correlation (86.6%) to the RNA-Seq data (Additional File 5); all but one of the qPCRs that failed to correlate did so because no difference was seen between the treatment and control samples. When the qPCR and RNA-Seq results differed, they often did so for more than one control-treatment pair, suggesting that the location or design of these primers may not accurately reflect mRNA accumulation, possibly due to variations in splicing.

### Transcript Analysis in Response to ABA and Osmotic Stress

**Figure 4:**
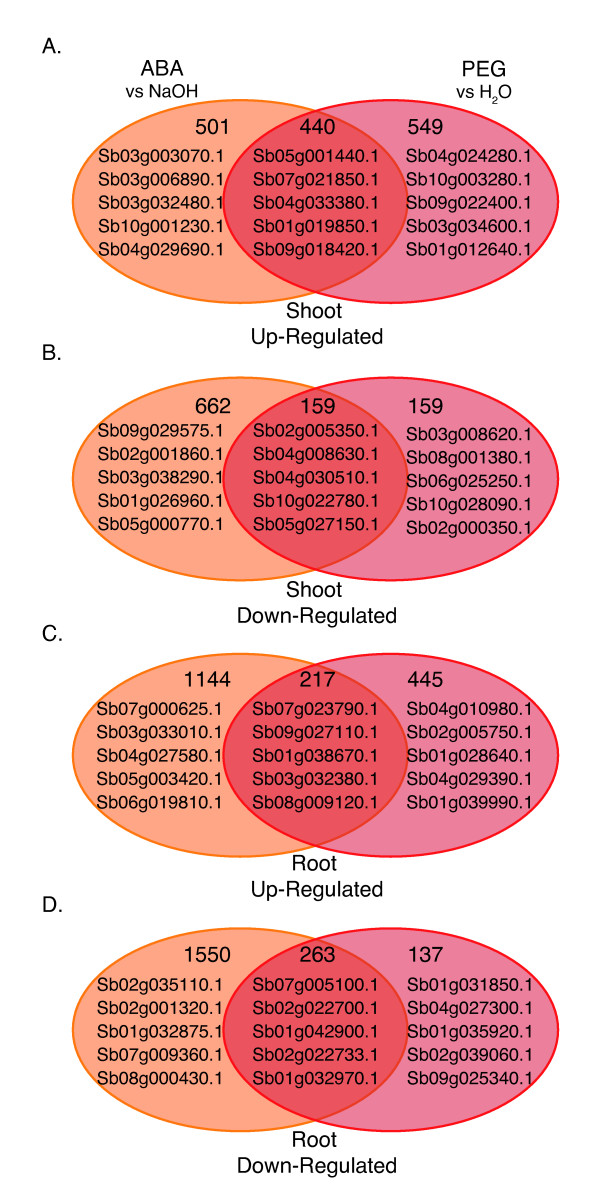
**Overlap between differentially responsive genes following treatment with ABA and PEG**. Venn diagrams display the overlap between differentially expressed genes following treatment with ABA (orange circles, right) and PEG (red circles, left) for 27 hrs in shoots (A, B) or roots (C, D). The total gene count for each category as well as the top five up- or down-regulated genes that exclusively fall into the given category are shown within each circle.

We explored the relative number of DE genes in roots and shoots in response to treatment with ABA or PEG (Figure 4). In general, fewer genes exhibited altered expression in response to osmotic stress than exogenous ABA treatment. Following the 27 hr ABA treatment, ~2,300 genes showed more than a 2-fold increase in gene expression whereas osmotic stress resulted in ~1,650 up-regulated genes (Figure 4A and 4C). Similarly, ~2,600 genes were down-regulated more than 2-fold in ABA-treated plants compared to ~700 genes in osmotically-stressed plants (Figure 4B and 4D). This is not surprising based on the involvement of ABA in response to plant stress, and its central role in other pathways, including dormancy in leaf [55-57], bud [55,58-65] and seed (reviewed in [66-68]). When comparing the overlap in DE genes between ABA and osmotic stress treatment, between 12-30% of the DE genes were in common between the two treatments depending on the tissue and whether gene expression was up- or down-regulated (Figure 4 and Additional File 3). When we consider the top five up- and down-regulated genes in response to ABA, PEG, or responding to both ABA and PEG treatment (Figure 4), we note that 29 out of 60 genes are considered to be uncharacterized or putative. As expected, genes similar to a late embryogenesis abundant (LEA) protein and a WSI18 protein, both of which are induced by water stress, and a dehydrin were in the top five genes up-regulated in response to both PEG- and ABA-treatment in roots and shoots, respectively. LEA proteins are hydrophilic proteins induced by drought stress and ABA, a subclass of which includes dehydrins (reviewed in [69-71]). Although their function is unknown, it has been suggested that LEAs act as water-binding molecules, membrane-stabilizers, and ion modulators (reviewed in [69-71]). A gene similar to OSIGBa010B08.10, whose gene product contains sugar substrate transporter domains was down-regulated in response to both ABA and PEG in roots, and a gene similar to peroxidase 6 was down-regulated in response to both treatments in shoots. Peroxidases comprise a large family of enzymes that function as antioxidants; as such, different peroxidases respond in various ways to drought stress [72], suggesting some family members, including Sb04g008630, may be down-regulated under water stress whereas others increase.

To further explore the genes responding to exogenous ABA and osmotic stress treatment (and their functions within the plant), gene ontology (GO) [73] and pathway enrichment analysis were performed. Enrichment analysis allows exploration of large datasets by suggesting that more genes fall within certain categories/pathways/groups than would be expected by a random draw. In total, 191 GO categories and 72 SorghumCyc pathways exhibited enrichment for DE genes based on significant p-values (GO analysis) or Z-scores plus p-values (pathway analysis) depending on the tissue/treatment combination being examined (Additional Files 6 and 7). Examples of GO categories exhibiting enrichment in DE genes included those involved in biotic and abiotic stress, cell growth and development, hormone biosynthesis, and sugar biosynthesis (Additional File 6). Similar to the GO analysis, numerous SorghumCyc hormone biosynthetic pathways showed enrichment in DE genes as did pathways involved in the biosynthesis of osmoprotectants, sugars, and amino acids, to name a few (Additional File 7).

While it is beyond the scope of this paper to discuss each GO category and metabolic pathway exhibiting DE gene enrichment, several examples of tissue- and/or treatment-specific enrichment are discussed in more detail below with particular emphasis on the role of DE genes in stress and defense response as well as possible influences on development. For those investigators desiring a broader overview of enriched GO categories and metabolic pathways, complete listings can be found in additional files 6 to 8.

### ABA and Osmotic Stress Pathways in Roots and Shoots

GO categories enriched for genes up-regulated in response to exogenous ABA or osmotic stress in roots and shoots include responses to light intensity, heat, wounding, and hydrogen peroxide (Additional File 6). Additionally, genes enriched in GO categories such as response to stress, cold, and water deprivation were also up-regulated in ABA and osmotic stressed roots and shoots, although in some treatment/tissue combinations, certain genes from these categories were down-regulated as well. ABA has been shown to play a role in the stress responses listed in these GO categories (reviewed in [35,38,74-76]), thereby supporting our results.

An example of a differentially expressed metabolic pathway common to both ABA treatment and osmotic stress includes the choline biosynthetic pathway, which was enriched for DE genes in roots and shoots based on GO and Z-score analysis (Additional Files 6 and 7). Choline can be oxidized to glycine betaine, a strong osmoprotectant [77,78]. In contrast to the choline biosynthetic pathway, we did not observe enrichment for the glycine betaine biosynthesis pathway based on the Z-score pathway analysis. This may be due to the fact that SorghumCyc pathways are computationally generated and populated by orthologous gene annotation (http://www.gramene.org/pathway/sorghumcyc.html).

As an alternate method to explore the influence of DE genes on pathway function, we analyzed the ratio of reactions in a given pathway that contain DE genes over all reactions within that pathway (Additional File 8). We note that out of the two reactions annotated in the glycine betaine pathway, at least one contained DE genes (data not shown). The altered regulation of the glycine betaine biosynthesis pathway is therefore still possible if a rate-limiting step is differentially expressed. A related osmoprotectant pathway, ß-alanine betaine biosynthesis, contained DE genes in all three reactions in the 4 tissue/treatment samples examined (Additional File 8). In support of this analysis method, the ß-alanine betaine expressing members of the Plumbaginaceae family have adapted to a wide variety of environments, including salt marshes and water-deficient locations, whereas glycine betaine producing members of the family have not [79].

Differential expression in amino acid metabolism pathways in response to osmotic stress and exogenous ABA was also indicated by analysis of SorghumCyc annotated pathways. The proline biosynthesis pathways contained DE genes in two out of three reactions assigned to the pathways for roots and shoots (Additional File 8). Proline is a known osmoprotectant employed by plants to enhance tolerance to abiotic stress, including drought. Proline can function as a reactive oxygen species scavenger, protect and stabilize proteins, and enhance the functions of certain enzymes (reviewed in [80-82]). In addition to the proline biosynthesis pathways, the majority of the reactions within the valine and leucine degradation pathways contain DE genes in response to exogenous ABA and osmotic stress (Additional File 8). Valine [83-86] and leucine [84-86] have been shown to accumulate in plants undergoing water stress, and their biosynthesis is auto-regulated by branched chain amino acid transferases (BCATs) as these enzymes control both the last step in biosynthesis and the first step in degradation ([86,87] and reviewed in [88]). Although we did not find differential expression of the BCAT homologs, it has been suggested that BCAT expression requires both dehydration and endogenous ABA, as exogenous application of ABA alone did not increase expression [84]. Although valine and leucine levels increase in Arabidopsis [84,86], tomato [85] and Bermuda grass [83], their turn-over rates have not been investigated. As the effect of the differential gene regulation on the accumulation of the products of the pathways, valine and leucine, is unknown, we suggest that the pathways are differentially regulated following ABA or osmotic stress treatment of sorghum tissues.

Several hormone biosynthetic pathways were also affected by exogenous ABA and osmotic stress in shoot and root tissue. Pathways for ethylene biosynthesis from methionine, gibberellin, JA, and brassinosteroid contain DE genes in 60% or more of their reactions (Additional File 8); some of these pathways also show enrichment based on Z-score pathway (Additional File 7) and GO analysis (Additional File 6), although not in every tissue/treatment combination. A relationship between water stress and ABA and GA has been reported in maize [34], soybean [33], and Arabidopsis [32,89]. Seo et al. [89] detail evidence, including increased expression of GA biosynthesis genes in the *aba2-2 *mutant, that supports possible regulation of GA metabolism by ABA, which is consistent with the differential expression of gibberellin biosynthesis genes in sorghum subjected to exogenous ABA and osmotic stress treatment.

Cytokinin degradation and conjugating pathways contain DE genes in every reaction within the pathway in all tissue/treatment samples (Additional File 8), although only roots treated with ABA or osmotic stress displayed enrichment for cytokinin conjugating pathways, and osmotically stressed shoots exhibited enrichment for cytokinin degradation by Z-score analysis (Additional File 7). Several types of cytokinin conjugations, including the creation of cytokinin glucosides, can render cytokinins biologically inactive. Studies conducted in tobacco have demonstrated that while the total amount of cytokinin rose under drought stress or ABA treatment, the majority of the cytokinin pool found within the roots was in the inactive form [90,91], suggesting an inverse relationship between ABA (drought) stress and active cytokinin accumulation. The present results support this hypothesis. Arabidopsis and tobacco plants over-expressing cytokinin-degrading cytokinin oxidase/dehydrogenase genes display increased root biomass as well as increased survival rates after water deprivation [92], reinforcing the link between decreased cytokinin levels and drought tolerance.

**Figure 5:**
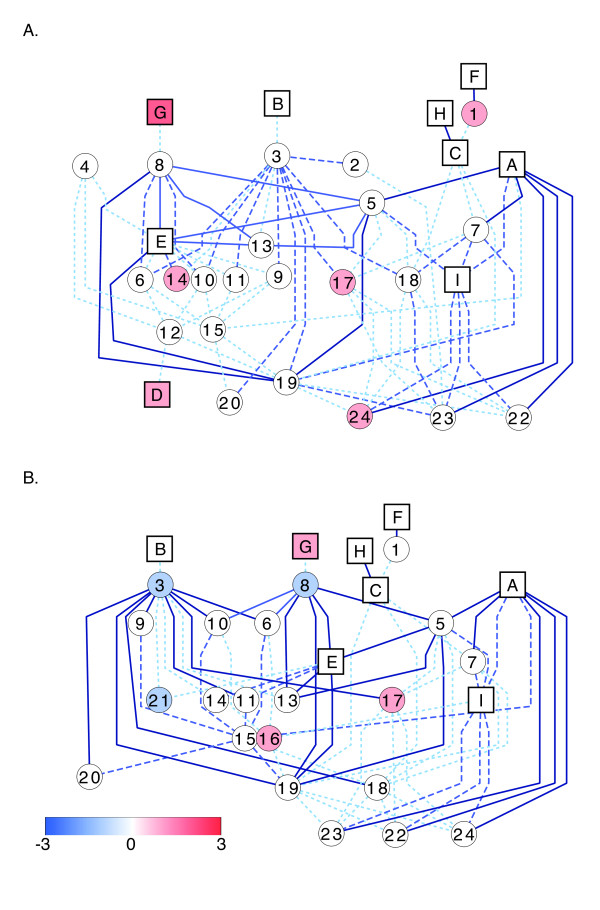
**Networks of hormone pathways in ABA-treated plants**. Networks were created considering the shortest paths connecting each hormone-related pathway to another hormone-related pathway in shoots (A) and roots (B). Hormone-related and non-hormone-related pathways are denoted as squares and circles, respectively, and are shaded based on the number of genes up-regulated within the pathway minus the number of genes down-regulated. Pathways that contain equal numbers of up- and down-regulated genes are white. Edges connecting the pathways occur only when differentially expressed genes are in common between the two pathways. Dark blue solid lines, blue long-dashed lines, and light blue short-dashed lines denote ≥10, 6-9, ≤5 DE genes, respectively, in common between the pathways. Pathway names are as follows: A, brassinosteroid biosynthesis; B, cytokinins degradation; C, cytokinins glucoside biosynthesis; D, ent-kaurene biosynthesis; E, ethylene biosynthesis from methionine; F, gibberellin biosynthesis; G, gibberellin inactivation; H, IAA conjugate biosynthesis; I, jasmonic acid biosynthesis; 1, anthocyanin biosynthesis; 2, ascorbate biosynthesis; 3, betanidin degradation; 4, Calvin cycle; 5, dTDP-L-rhamnose biosynthesis; 6, fructose degradation to pyruvate and lactate; 7, galactose degradation; 8, γ-glutamyl cycle; 9, gluconeogenesis; 10, glycolysis; 11, methionine biosynthesis; 12, oleoresin sesquiterpene volatiles biosynthesis; 13, oxidative ethanol degradation; 14, phenylalanine biosynthesis; 15, phenylpropanoid biosynthesis; 16, ribose degradation; 17, starch biosynthesis; 18, sucrose degradation; 19, sucrose degradation to ethanol and lactate; 20, threonine biosynthesis from homoserine; 21, triacylglycerol degradation; 22, UDP-galactose biosynthesis; 23, UDP-glucose conversion; 24, UDP-N-acetylgalactosamine biosynthesis.

**Figure 6:**
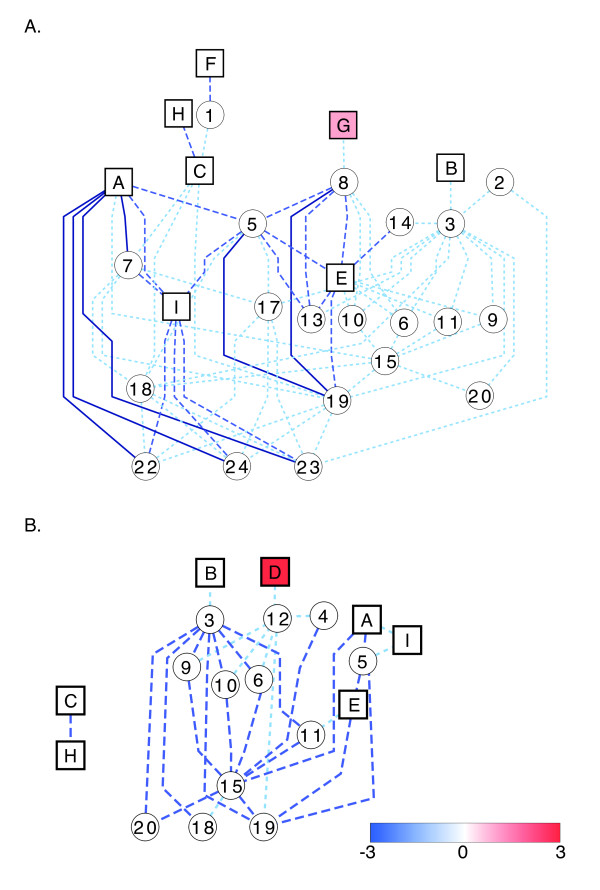
**Networks of hormone pathways in PEG-treated plants**. Networks were created considering the shortest paths connecting each hormone-related pathway to another hormone-related pathway in shoots (A) and roots (B). Hormone-related and non-hormone-related pathways are denoted as squares and circles, respectively, and are shaded based on the number of genes up-regulated within the pathway minus the number of genes down-regulated. Pathways that contain equal numbers of up- and down-regulated genes are white. Edges connecting the pathways occur only when differentially expressed genes are in common between the two pathways. Dark blue solid lines, blue long-dashed lines, and light blue short-dashed lines denote ≥10, 6-9, ≤5 DE genes, respectively, in common between the pathways. Pathway names (A-I and 1-24) are denoted as in Figure 5.

Cross-talk between hormone pathways via associated genes is becoming a common realization in plants, especially in leaf tissue ([32,34,89] and reviewed in [93,94]). Considering a network based on SorghumCyc hormone pathways and their neighbor pathways (pathways that contain overlapping DE genes with those found in the hormone-related pathways) (Figures 5 and 6), we observe that few of the hormone pathways share overlapping DE genes. Only the brassinosteroid and JA biosynthesis pathways, and cytokinin glucoside and IAA conjugate biosynthesis pathways are directly connected via DE genes (Figures 5 and 6, squares labeled A and I, and C and H, respectively). The lack of overlapping DE genes within the hormone pathways suggests that if hormone levels are changing as a result of alterations at the transcriptional level, they do so in a non-concordinate manner. Taken together, our data support indirect 'cross-talk' between the various hormones in response to osmotic stress and ABA. As many of the pathways contain both up- and down-regulated genes often in equal number (Figures 5 and 6; white circles and squares), the effect of differential gene expression on the pathway outputs cannot be predicted from the present results.

### Defense Pathways are Regulated by ABA and Osmotic Stress in Shoots

The 13-lipoxygenase (13-LOX) and 13-HPL pathways as well as the divinyl ether biosynthesis II pathway were enriched in DE genes in shoots following treatment with either ABA or osmotic stress (Additional File 7). In plants, the most common LOX substrates, linoleic and linolenic acids, are converted into a variety of bioactive mediators involved in plant defense, senescence, seed germination, and plant growth/development ([95,96] and reviewed in [97-99]). ABA is also found to increase 13-LOX genes in maize leaves [100], supporting a common role for the 13-LOX pathways in ABA and osmotic stress response in maize and sorghum. Two 13-LOX genes in maize have been characterized and display distinct transcriptional patterning in response to ABA as exhibited by differing peak response times, as well as induction of only one of the genes in response to cold, a stress response in which ABA plays a role [100]. GO analysis revealed enrichment for DE genes in defense response, and abiotic stresses including heat, osmotic, and reactive oxygen species (Additional File 6) for both 27 hr ABA- and osmotic stress-treated shoots. In Arabidopsis [101], a shift towards a general abiotic stress response was also observed 6+ hrs after stress induction in shoot tissues similar to what we observe in sorghum.

### ABA and Osmotic Stress Treatments Mimic Root Disease Response

Examination of GO categories displaying DE genes in roots in response to ABA or osmotic stress revealed a series of pathways that have been implicated in a plant's response to pathogen attack [102-107]. GO categories enriched in up-regulated DE genes in osmotically stressed roots and down-regulated in ABA-treated roots included cell wall modification and cell growth, response to nitrate, JA stimulus, SA stimulus, and hypersensitive response. By comparison, the GO category hydrogen peroxide catabolism was enriched for genes down-regulated in osmotically stressed and ABA-treated roots (Additional File 6). Cell wall modification and oxidative stress response has been suggested to be regulated by JA [106], and JA treatment has been shown to decrease the transcriptional activity of genes which respond to ABA leading to an antagonistic relationship between the two hormones [106]. Salicylic acid and ABA have antagonistic roles in plant defense ([104,105] and reviewed in [107]); SA is induced in plants under pathogen attack, and the ABA biosynthetic pathway is induced in the plant by the pathogen to reduce plant response (reviewed in [107]). Salicylic acid and JA enhance biotrophic and necrotrophic resistance, respectively, with possible cross-talk between the two hormones (reviewed in [102,107]). Salicylic acid, JA, and ABA are complexly related in pathogen response, which is normally accompanied by an oxidative burst of reactive oxygen species (ROS) (reviewed in [102,103]). Peroxides are considered to be pathogen responsive and remove hydrogen peroxide from the cell (reviewed in [103]). Intriguingly, they also increase cell wall rigidity by cross-linking cell wall components (reviewed in [103]). It remains to be determined whether the GO categories highlighted in this cascade of events are solely due to a side effect of altered hormone levels due to osmotic stress or are part of a survival mechanism activated in water-limited conditions.

### Remodeling and Growth in PEG-Treated Roots

A number of GO categories involved in growth and remodeling exhibited specific enrichment in roots following osmotic stress treatment. For example, up-regulated genes in PEG-treated roots were enriched in the GO categories for nodulation and regulation of epithelial cell differentiation, whereas down-regulated genes were enriched in the GO categories of lateral root development and nitrate transport (Additional File 6). These results support a well-established role for root remodeling in response to osmotic stress [108-110]. Genes down-regulated in response to osmotic stress display enrichment for auxin efflux, phosphate transport, and lateral root development (Additional File 6). Low phosphate increases lateral root development and reduces primary root elongation through altered auxin distribution [111-116], suggesting an alteration in root architecture in osmotic-stressed roots. Enhanced root growth and drought tolerance were associated with reduced cytokinin levels in Arabidopsis and tobacco [92], and we observed enrichment for cytokinin glucoside biosynthesis in roots treated with PEG (Z-score > 3; Additional File 7), suggesting that osmotic stress may play a role in root remodeling and growth in sorghum.

### Transcriptional Regulation of Differentially Expressed Genes

ABA-responsive elements (ABREs) [117], dehydration-responsive elements (DREs) [118], and low-temperature-responsive elements (LTRE) [119,120] are known to regulate gene expression in response to ABA, drought, and cold stress, respectively. Given that genes in our DE lists were alternatively expressed due to the presence of ABA or osmotic stress, we searched 1000 bp upstream of all sorghum genes for the presence of *cis*-acting elements found within PlantCARE (http://bioinformatics.psb.ugent.be/webtools/plantcare/html/) [121,122] and PLACE (http://www.dna.affrc.go.jp/PLACE/index.html) [123,124], and highlighted those elements that are known to be involved in ABA and drought response and those that support the models of gene interaction supported by our GO and SorghumCyc analysis.

Transcription factor analysis revealed that the AP2-EREBP TF family is over-represented in DE genes from shoots following ABA treatment (p-value < 0.02). The AP2-EREBP TF family consists of several subgroups including dehydration-responsive element binding proteins (DREBs), ethylene response factors (ERFs), APETELA2 (AP2)-related proteins, as well as those related to ABI3/VP1 (RAVs), which contain both AP2 and B3 motifs (reviewed in [125]). Genes up-regulated in shoots following ABA-treatment are also enriched for *cis*-elements including the dehydration-responsive elements DRE2COREZMRAB17 and DRECRTCOREAT, as well as RAV1BAT, a binding site for RAV1, a member of the AP2-EREPB TF (Additional File 9). AP2-EREBP TF family members play roles in response to both hormones (ABA) and drought stress (reviewed in [125]), and our *cis*-element enrichment analysis supports similar claims in sorghum.

Sugar-repressive motifs were enriched in genes down-regulated in shoots by ABA and osmotic stress (Additional File 9). Sugar production increases in Arabidopsis, rice and other plants exposed to osmotic stress (reviewed in [74,126-130]), and enrichment of sugar-repressive motifs in genes down-regulated by osmotic stress is consistent with this observation.

Although several of the same *cis*-elements are enriched across all samples, few transcription factors are highly expressed in common between roots and shoots after exogenous ABA- and osmotic stress treatment. A small set of 6 TFs are up-regulated in all tissue/treatment combinations that include bZIP - Sb04g034190; C3H - Sb09g006050, Sb03g003110; HSF - Sb03g033750, Sb10g021800; and MYB-related - Sb03g003100 (Additional File 3). ABF2 (AREB2), the Arabidopsis homolog of Sb04g034190 (Additional File 3), is up-regulated in response to drought [131] and ABA-treatment and plays a role in glucose response as well as salt, heat, and oxidative stress tolerance [132]. *At*OZF1 (At2g19810) is the homolog to both Sb09g006050 and Sb03g003110 (Additional File 3) and *AtOZF1 *mRNA accumulation is enhanced when seedlings are exposed to ABA, salt, and hydrogen peroxide [133]. *At*OZF1 has recently been shown to localize in or attach to the plasma membrane and improve oxidative stress resistance by enhancing the transcription of cytosolic *ASCORBATE PEROXIDASE 1 *(*APX1*) and *GLUTATHIONE S-TRANSFERASE TAU 5 *(*AtGSTU5*), two antioxidant enzymes [133]. The functions of homologs to Sb03g033750, Sb10g021800, and Sb03g003100 have not been investigated.

Combining information from PlnTFDB [134,135], PlantTFDB [136-138], and GrassTFDB [139], sorghum contains 79 TF families and 2202 unique genes, 95 of which are assigned to more than one TF family. In examining specific tissue/treatment combinations, ABA-treated roots displayed the greatest change in TFs with expression of 101 up-regulated and 112 down-regulated genes (Additional File 3). Approximately 50% of the differentially expressed TFs, whether up- or down-regulated, were specific for a given tissue/treatment combination (Additional File 3). The AP2-ERF family of TFs is the most altered in expression when sorghum seedlings are exposed to ABA or osmotic stress (data not shown). This family of TFs has been shown to respond to a diverse array of biotic and abiotic stresses in rice [140]. Furthermore, the overexpression of members of this TF family in rice [141], *Trifolium alexandrinum L. *[142], and tobacco [143] resulted in an increased tolerance to salt and drought in transgenic plants.

### Gene Products of Unknown Function across Plant Species are Differentially Expressed in Response to Abiotic Stress

**Figure 7:**
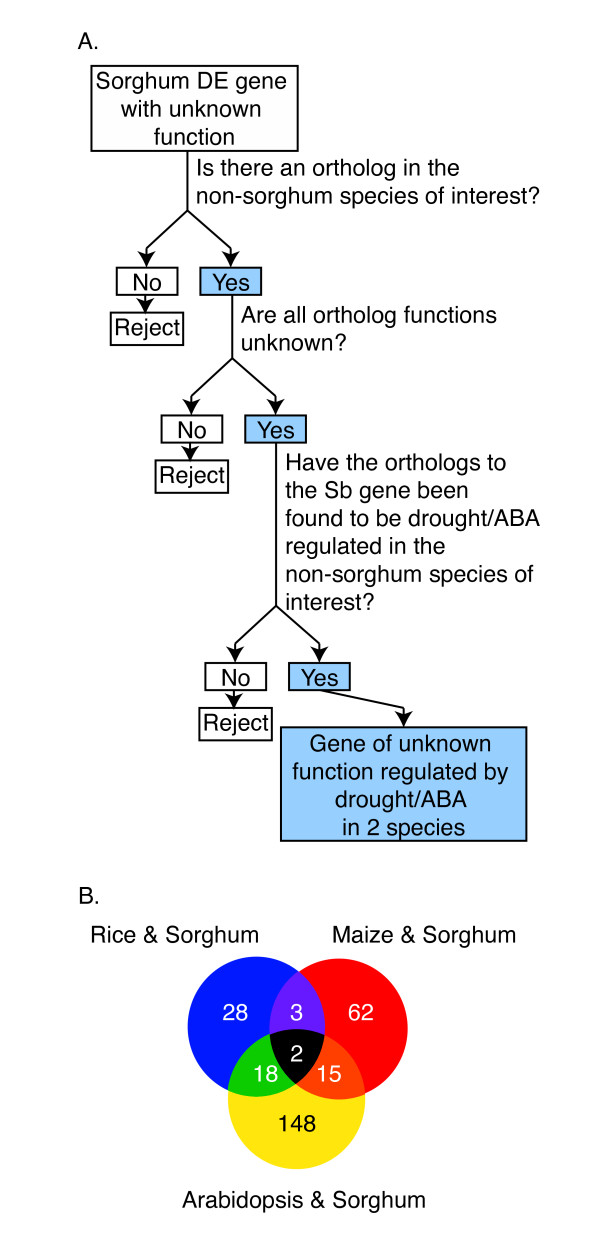
**Determining the genes of unknown function that respond to drought or ABA treatment across species**. Decision tree used to determine which genes and their orthologs were regulated by drought/ABA across different species (A). For each sorghum gene, the tree was traversed 3 times; once for each non-sorghum species: rice, maize, Arabidopsis. Venn diagram displaying the overlap of drought-responsive sorghum genes of unknown function that had drought-responsive orthologs of unknown function in other species (B). Each gene is found in 2 or more species.

Given the advances in genomic technology platforms, the unique ability to compare transcriptomes across several species can be exploited to cross-reference information concerning genes and gene function. Lists of alternatively expressed genes in rice [24,26,144,145], maize [30], and Arabidopsis [13,15,17] under various forms of drought stress have been published. Many of these DE genes were of unknown function. We hypothesize that orthologs exist between genes of unknown function across species, and that a subset of these orthologs will be differentially expressed in response to abiotic stress. We compared orthologs of sorghum, rice, maize, and Arabidopsis (http://www.gramene.org) [146], cross-referenced published drought-responsive species-specific gene lists [13,17,26,30,144,145], and identified genes of unknown function (Figure 7A). We were interested in genes of unknown function that respond differentially under osmotic stress and therefore did not filter our results based on directionality of the gene expression in response to drought as the published gene lists were created under various severities of drought [30], using different technologies [13,17,26,30,144,145], and the species considered respond differently to water-limited conditions. When sorghum DE gene lists were compared with the sorghum orthologs of published drought responsive gene lists from rice, maize, and Arabidopsis, we discovered 51, 82, and 183 genes, respectively, of unknown function that were drought responsive (Figure 7B and Additional File 10). Two sorghum genes, Sb01g045990 and Sb03g005990, and their orthologs were differentially expressed in all four species (Figure 7B and Additional File 10).

To investigate these DE genes further, we searched for stress-responsive *cis*-acting promoter elements by scanning 1000bp upstream of orthologous genes. The 51 sorghum genes orthologous to unknown rice genes responsive to drought were enriched for ABREs and CGTCA-motifs; motifs that are involved in responses to ABA [147] and methyl jasmonate [148], respectively (Additional File 11). The promoters from the rice orthologs show enrichment for both SORLIP1AT and ABRE motifs (Additional File 12). The 183 drought-responsive sorghum genes with drought-responsive orthologs of unknown function in Arabidopsis are enriched for Box S, involved in the wounding and pathogen response [149], CCAAT-box motifs, possible binding sites for MYB proteins [150], and ABREs (Additional File 11). The promoters for the Arabidopsis orthologs are enriched for a circadian clock motif, which is involved in circadian mRNA accumulation [151] (Additional File 12). The 82 sorghum genes with orthologs in maize with unknown function and responsiveness to drought are enriched in ABREs, Box S, DRECRTCOREAT, a dehydration responsive element [152,153], as well as, CBFHV and LTRECOREATCOR15 [119,120], which are both involved in low temperature response (Additional File 11). The corresponding maize ortholog promoters are enriched for the TGA-1 motif, a known auxin-responsive element [154,155] (Additional File 12). Due to the conservation of these presently unknown gene products, our results suggest that these elements play an important and ecologically conserved role in the response to water-limiting environments.

## Conclusions

The present study demonstrates the value of whole-genome transcriptome analysis generated by RNA-Seq for accurate quantification of gene expression on a genome-wide scale. Through mapping more than 689.5 million sequence reads, we established transcriptome data sets for sorghum subjected to osmotic stress, exogenous ABA, or control conditions. Moreover, mined in parallel with existing RNA-Seq resources from other species, this expression compendium provides a powerful resource for cross-species comparisons of gene expression and function. Thus, our initial analysis provides insight into how osmotic stress and hormonal treatment alter gene expression in this drought-tolerant cereal species, and has facilitated an initial assemblage of *cis*-regulatory elements and transcription factors working in union to alter gene expression in response to these conditions.

Concomitantly, we expanded on the previous sorghum cDNA-array differential gene expression study of Buchanan et al. [31] by examining the expression of all annotated protein and non-coding genes, while concomitantly refining the methodology used to quantify gene expression. Sequencing of the sorghum genome [43] and development of SorghumCyc allowed for exploration of differentially expressed pathways, and an initial glimpse into the intricate involvement of metabolic pathway cross-talk in the cellular response to abiotic stress. The sorghum RNA-Seq data set represents a community-wide resource that will allow scientists to query gene expression and annotation, and provides an initial look at the cascade of global gene expression patterns that occur in response to water-limiting environments.

## Methods

### Plant Materials and Growth Conditions

Seeds of *Sorghum bicolor *genotype BTx623 were germinated by soaking in distilled, aerated water overnight, and then transferred to moist germination paper for 3 days. The seedlings were then transferred to 0.5× Hoagland's solution and allowed to grow for an additional 5 days in a controlled environment chamber with a day length of 12 hrs, 50% humidity, and day/night temperatures of 31°C/22°C, respectively. At 8 days, the plants were treated with 20 μM ABA ((+/-)-*cis*, *trans*-abscisic acid (Sigma, St. Louis, MO) dissolved in NaOH), 57.1 μM NaOH (control for ABA treatment), 20% PEG-8000 (Sigma, St. Louis, MO), or milliQ water (PEG control) by adding these solutions to the Hoagland's solution for 27 hours; a time point chosen to mimic the parameters of Buchannan's treatment of the plants prior to cDNA microarray analysis [31].

Three groups of 10 plants each were harvested post-treatment as paired shoots and roots; the roots and shoots sequenced in each run are two parts from the same plants. Shoot and root tissue were quickly divided at the residual seed coat, flash frozen in liquid N_2 _and stored at -80°C until used for RNA extraction. The hydroponic experiment was conducted three times over the course of several months in this manner with each experiment representing one biological replicate.

### RNA Extractions and Pooling

Total RNA was extracted using the miRNEasy kit (Qiagen, Valencia, CA) from paired shoot/root samples; one sample per bucket per treatment per hydroponic experiment (Figure 1). Three equimolar RNA samples for a particular treatment from a single hydroponic experiment were combined and used to prepare cDNA for RNA-Seq analysis as described below.

### RNA-Seq cDNA Preparation

cDNA was generated using the RNA-Seq kit according to the manufacturer's protocol (Illumina Inc., San Diego, CA). In short, poly-A RNA was isolated from total RNA and chemically fragmented. First and second strand synthesis were followed by end repair, and adenosines were added to the 3' ends. Adapters were ligated to the cDNA and 200 ± 25 bp fragments were gel purified and enriched by PCR. cDNA was quantified using the Qubit BroadRange Assay (Invitrogen, Carlsbad, CA), checked on a Bioanalyzer 2100 (Agilent Technologies, Santa Clara, CA) and run on the Illumina GAIIx Sequencer (Illumina Inc.) using version 4 reagents. Single-read sequences of length 51 bp were collected and have been deposited in GEO (GSE30249).

### Analysis of RNA-Seq Sequences

Base calling on the GAIIx was performed by Illumina's Real Time Analysis (RTA) software to produce sequence files that were used for alignment and the determination of gene counts. Sequences were trimmed to 50 bp and aligned to the sorghum genome (sbi1 downloaded from ftp://ftp.jgi-psf.org/pub/JGI_data/phytozome/v6.0/Sbicolor/assembly/) using the ELAND_rna algorithm and raw counts per gene returned using CASAVA v1.6 software according to the Illumina CASAVA1.6 User Guide. ELAND (Efficient Large-Scale Alignment of Nucleotide Databases) searches a set of large DNA files for a large number of short DNA reads allowing up to 2 errors per match. ELAND_rna required additional files to analyze RNA sequencing data including an abundant sequences file that contained sorghum chloroplast, mitochondria and repeat sequences, an exon coordinates file and a splice junctions file (these last two files were generated from Sbi1.4.gff3 downloaded from ftp://ftp.jgi-psf.org/pub/JGI_data/phytozome/v6.0/Sbicolor/annotation/ using a combination of custom perl scripts and those provided with the CASAVA v1.6 package). All of the sorghum files necessary for alignment and determination of gene counts using the CASAVA V1.6 software are available upon request. edgeR [48-50] was used to simultaneously quantile normalize the raw counts for each run due to an upgrade to the Illumina RTA software (from v1.5 to v1.6) that resulted in higher sequence counts for our third biological replication. Quantile normalization for all Illumina runs was conducted once to allow for comparison across all samples, as described below. The raw counts from the output of CASAVA v1.6 were given to the edgeR package (version1.4.7) in R [48-50] and quantile normalized together within edgeR functions. edgeR finds changes between two or more groups when either or both groups contain replicates, by using a negative binomial distribution model and estimating genewise dispersions based on conditional maximum likelihood [48-50]. In edgeR, an appropriate object (d) was created by calling the DGEList function parameters as follows: counts = matrix of raw counts, group = list of order of columns in counts, lib.size = NULL, remove.zeros = TRUE. A common dispersal of the counts was rendered using the estimateCommonDisp function on the previously made object (d). Next, analysis was performed on the desired pairs using the exactTest function (de.com). The genes found to be differentially expressed and with a p-value of less than 0.01 were retrieved using the function topTags; parameters used were de.com and n = sum(de.com$table$p.value < 0.01)). Taking into consideration the 3 biological replicates for the pairs of samples (i.e. treatment and control), edgeR returned a list of differentially expressed genes, which were then filtered for a p-value adjusted by multiple-hypothesis testing of less than 0.05. Gene list results were filtered for genes with a median sequencing depth of 2X in at least one of the two samples compared (i.e. control or treatment) and a log_2_-fold change of ≥ 1 or ≤ -1. Gene counts were not normalized for gene length, as we did not compare expression levels across genes, but rather expression across treatments.

### Determination of Read Depth Cutoff Value for Differential Gene Expression

Trends in the data were examined to identify an appropriate cutoff value for determination of differential gene expression. Approximately 40% of all expressed sorghum genes had between 5,001-50,000 mapped RNA-Seq reads (Additional File 13), and ~30% were sequenced to a depth of 10-50X (Table 2). The distribution of read depth per gene was comparable across all runs with >80% of transcriptionally active genes having more than 500 read counts (Additional File 13). At a read depth of 2X, 13,964 genes were expressed in at least one tissue/treatment combination (data not shown). Of the 26,466 annotated gene models detected in the present study with median sequencing depth of greater than zero (i.e. read counts for a gene were 0 for at most 1 run), 2,971 were expressed at a median read depth of less than 2X but greater than 0.5X in at least one tissue/treatment combination (data not shown). Thus ~11.2% of the sorghum genes detected in this study either exhibit low expression in the present experimental conditions tested or represent background signal in the RNA-Seq method. Therefore, to minimize false positives while retaining genes of lower expression, a 2X median read depth cutoff in one of the two samples being compared (i.e., ABA-treated vs. NaOH-treated) was chosen for examination of differential gene expression.

**Table 2:** Number of Gene Models Binned by Median Sequencing Depth Cut-off

	**0x SD^a^**	**≤ 1x SD**	**1x < SD ≤ 2x**	**2x < SD ≤ 5x**	**5x < SD ≤ 10x**	**10x < SD ≤ 50x**	**> 50x SD**
	
ABA-Treated Roots	10004	5136	2026	3599	3816	7542	2022
NaOH-Treated Roots	9943	5158	2110	3625	3694	7547	2068
PEG-Treated Roots	9674	5154	1987	3620	3792	7753	2165
H_2_O-Treated Roots	9925	5189	2115	3627	3673	7558	2058
ABA-Treated Shoots	10639	5695	2072	3641	3784	6424	1890
NaOH-Treated Shoots	10462	5642	2053	3553	3736	6625	2074
PEG-Treated Shoots	10033	5465	2058	3533	3776	7249	2031
H_2_O-Treated Shoots	10794	5800	2060	3647	3804	6251	1789

^a^SD denotes sequencing depth calculated as the total number of bases mapped to a gene (exons only) divided by total gene (exon) length.

### qPCR Validation

For each sample, 10 μg total RNA was treated with Turbo DNA-*free *(Ambion, Austin, TX), 1 μg of which was reverse transcribed in a 20 μL volume using 200 units SuperScript III (Invitrogen) primed with random hexamers. The resulting cDNA was diluted to 200 μL with water. Gene-specific primers were designed to span the last exon of the transcript using Primer3 software (http://frodo.wi.mit.edu/primer3/). Quantitative real time PCR was carried out in triplicate using the ABI Prism 7900HT Sequence Detection System on 1 μL diluted cDNA, *Power *SYBR Green PCR Master Mix (Applied Biosystems, Carlsbad, CA) and primers at a final concentration of 0.05 μM each. Primer sequences are contained in Additional File 5. The 18S primers and probe (TaqMan Ribosomal RNA Control Reagents kit, Applied Biosystems) were used at a final concentration of 0.025 μM, with 1 μL diluted cDNA and TaqMan Universal PCR Master Mix (Applied Biosystems). PCR conditions were 2 min at 50°C and 10 min at 95°C followed by 47 cycles of 95°C for 15 sec and 60°C for 1 min and the dissociation period of 95°C for 15 sec, 60°C for 15 sec and 95°C for 15 sec. DNA amplification was monitored in real time using ABI Prism 7900HT Sequence Detection System software (v2.2). Amplification of 18S rRNA was monitored as an endogenous control that was used to normalize template amounts. Control reactions in which reverse transcriptase was omitted did not give amplification signals above the threshold.

### SorghumCyc Pathway Enrichment Analysis

SorghumCyc is a pathway/genome database that integrates genomic information with experimentally elucidated and electronically derived functional annotations to infer metabolic pathways in sorghum [146]. Using Z-score statistics, a method to determine how many standard deviations a given observation is from the standard mean, we performed an enrichment analysis on SorghumCyc data (ftp://ftp.gramene.org/pub/gramene/pathways/sorghumcyc (ver 1.0 beta)). Data were filtered such that gene models thought to occur on the sorghum scaffolds (name format Sb###s######) were removed. The Z-score was calculated as the quantity of the number of observed counts minus the expected counts, divided by the square root of the standard deviation of the expected counts for each pathway. The observed counts are defined as the number of DE genes within a pathway. The expected counts are determined by multiplying the number of genes in the DE gene list of interest by the number of genes within the pathway of interest and dividing this value by the number of unique genes in the collection of all pathways. SorghumCyc pathways were considered significantly enriched if the following criteria were met: Z-score ≥ 2, p-value ≤ 0.05, and the expected number of genes for a family > 1. Pathways within SorghumCyc are conveniently broken into reactions using data from SorghumCyc source files (*i.e.*, pathways.dat, reactions.dat, enzrxns.dat, proteins.dat, and genes.dat). The ratio of reactions in a given pathway containing DE genes divided by the number of reactions within the pathway was then determined and considered an alternate method for determining possible pathway regulation.

### GO Enrichment Analysis of Differentially Expressed Gene Sets

GO enrichment was determined using the goseq package [156] in R using annotations from agriGO (http://bioinfo.cau.edu.cn/agriGO/) [157]. The agriGO genes-GO annotation pairs were filtered such that gene models thought to occur on the sorghum scaffolds (name format Sb###s######) were removed; gene models were condensed by removing the number trailing the "." and retaining only unique gene-GO annotation pairs. Each unique gene within a GO category was allowed to contribute to the enrichment of that category, regardless of the number of categories it is annotated to. goseq compensates for selection bias (i.e. length of the genes within different categories) and determines significance based on an extension of the hypergeometric distribution (Wallenius non-central hypergeometric distribution) [156]. Categories were considered significant if the p-value ≤ 0.05.

### Transcription Factor Analysis

Transcription factor information and protein sequences were downloaded from PlnTFDB (http://plntfdb.bio.uni-potsdam.de/v3.0/) [134,135]. The protein sequences were blasted against the sorghum genome to determine the JGI accepted gene identification; the top hits with e-value less than 0.1 were retained. Transcription factors from PlnTFDB were combined with those from PlantTFDB (http://planttfdb.cbi.pku.edu.cn/download/gene_model_family/Sbi) [136-138] and GrassTFDB (http://grassius.org/browsefamily.html?species=Sorghum) [139]. Annotations from members of the same TF family were collapsed if the names were the same, but formatted differently (e.g. JUMONJI and Jumonji or MYB-related and MYB_related). Enrichment analysis was performed using Z-score analysis as defined above. Transcription factors were considered significant if the following criteria were met: Z-score ≥ 2, p-value ≤ 0.05, and the expected number of genes for a family > 1.

### *cis*-Acting Promoter Element Analysis

Promoter elements were downloaded from PlantCARE (http://bioinformatics.psb.ugent.be/webtools/plantcare/html/) [121,122] and PLACE (http://www.dna.affrc.go.jp/PLACE/index.html) [123,124]. These databases were used as they allowed for easily downloadable, queryable, and manipulatable files that could be used to run an analysis in-house. The element names were collapsed if the sequences for them were identical. Promoter sequences (1000 bp) for all sorghum genes were determined based on the Sbi1.4 annotation and downloaded from Gramene BioMart (http://gramene.org/biomart/martview). As current annotation is not refined, some genes lack complete 5'-UTR lengths, and, for these genes, the 1000 bp upstream sequences analyzed will contain partial or complete 5'-UTR sequence. *cis*-elements were located in the upstream regions by matching in R; location and number were recorded. Enrichment was considered significant if the following criteria were met: Z-score ≥ 2, a hypergeometric distribution adjusted p-value of ≤ 0.05, and the expected number of genes for a family > 1.

### Ortholog Analysis

Gene descriptions and putative ortholog pairs from the Arabidopsis, rice, sorghum and maize genomes were derived from Gramene BioMart (http://gramene.org/biomart/martview) [146]. Manipulations were performed in R. The method is summarized in Figure 7. Sorghum DE genes were filtered for those with unknown function by searching for the terms 'hypothetical', 'expressed', 'predicted' and 'uncharacterized'. Using the data from Gramene, we determined if these genes contained orthologs in the species of interest and whether all orthologs designated for a particular gene were of unknown function. This smaller listing of genes was then assayed for previously published drought responsiveness in the non-sorghum species. The resulting *Sb*-ortholog pairs are of unknown function in sorghum and the non-sorghum species of interest and responsive to drought in both species. We did not require that the gene expression across the different species lists occur in the same direction, only that the genes were DE, as genes within single species can range in expression from repression to induction depending on the severity of the drought stress [30].

### Network Analysis

Network analysis was performed in R using SorghumCyc pathways (http://www.gramene.org/pathway/sorghumcyc.html) [146] that had been manually collapsed. This collapse consisted of combining related pathways (e.g. alanine biosynthesis II and III or cytokinins *7-N*-glucoside, cytokinins *9-N*-glucoside, and cytokinins-*O*-glucoside biosynthesis). The pathways were filtered such that only genes of interest were considered as edges between nodes (pathways). Matrices were generated that contained the paired pathway names and the number of DE genes in common between them. The information was then imported into Cytoscape [158,159] to visualize the networks.

## Authors' contributions

DVD generated the RNA-Seq cDNA and DE gene lists, performed *cis*-element, GO, SorghumCyc pathway, and transcription factor analysis, and drafted the manuscript. MKM contributed to the SorghumCyc pathway analysis and reviewed the manuscript. AO developed alternative scripts to process RNA-Seq data and compare the results to sorghum genomic annotations and reviewed the manuscript. RRK contributed to the interpretation of the data and helped draft the manuscript. SK performed GO enrichment analysis, retrieved gene descriptions and putative ortholog pairs, and reviewed the manuscript. DW contributed to the experimental design, interpretation of the data, and helped draft the manuscript. PEK contributed to the experimental design, processed the RNA-Seq data to generate raw counts per gene, contributed to the interpretation of the data, and helped draft the manuscript. All authors read and approved the final manuscript.

## Supplementary Material

Additional file 1 **Lane by lane summary of RNA-Seq data**. ^†^Read mapping to splice junctions included within the total number of reads mapped to genes. Click here for file

Additional file 2 Pearson correlation coefficients for roots and shoots across all runs Click here for file

Additional file 3 **Differentially expressed genes in treatment vs. control roots and shoots based on a 2X sequencing depth**. ^a^Confidences refer to those assigned to the gene models by Patterson et. al [43]. ^b^ABI3VP1 - proteins containing a B3 domain and named after the founding members (*ABA INSENSITIVE 3 *and *VIVIPAROUS1*); AP2 - Apetela2-like proteins that contain one repeated AP2/ERF domain; AP2-EREBP - Apetela2 and Ethylene-responsive element binding proteins containing two repeated AP2/ERF domains; ARF - auxin response factor family members; ARR-B - members of the type-B phospho-accepting response regulator family; AUX/IAA - auxin/indole-3-acetic acid family members controlled by auxin-responsive elements (AuxREs); B3 - Superfamily that encompasses the auxin response factor family, and the LAV, RAV and REM family containing an ~110 amino acid region called the B3 domain; bHLH - basic helix-loop-helix protein; bZIP - basic leucine zipper protein; C2C2-CO-like - similar to "CO-like"; C2C2-Dof - proteins containing DNA-binding with one finger domain and a highly conserved DNA-binding domain, which includes a single C2-C2 zinc finger; C2C2-GATA - GATA-binding proteins containing one or two highly conserved zinc finger DNA-binding domains; C2C2-YABBY - proteins containing a C2C2 zinc finger-like domain towards the amino terminus and a helix-loop-helix (YABBY) domain; C2H2 - proteins containing zinc finger domains with a secondary structure stabilized by a zinc ion bound to the Cys and His residues of the finger; C3H - proteins containing a Cys3His zinc finger domain; CCAAT - proteins contained within the NF-A complex that recognize CCAAT box motifs; CCAAT-HAP2 - HAP2 proteins of the heterotrimeric CCAAT-box-binding complex (HAP2, HAP3, and HAP5); CO-like - CONSTANS-like proteins, containing both a zinc-finger and CCT (CO, CO-like, TOC1) domain; CPP - cysteine-rich polycomb-like proteins containing one or two Cys-rich domains; DBB - proteins containing double B-box zinc finger domains; Dof - proteins containing DNA-binding with one finger domain; EIL - EIN-3-like transcription factors involved in ethylene signaling; ERF - ethylene-responsive factors; FAR1 - far-red-impaired response family members; G2-like - proteins similar to G2 (maize); GATA - proteins that interact with conserved WGATAR; GeBP - proteins similar to GL1 enhancer binding protein and containing a central region with no known motifs and a C-terminal region with a putative leucine-zipper motif; GNAT - Gcn5-related N-acetyltransferase superfamily members; GRAS - named for GAI, RGA, SCR family members; GRF - proteins containing the same QLQ and WRC domains found in G*ROWTH-REGULATING FACTOR1 *(*GRF1*); HB - similar to "Homeobox"; HD-ZIP - proteins containing a homeodomain and leucine zipper motif; HMG - proteins containing high-mobility-group boxes initially identified as DNA-binding domains; Homeobox - homeobox proteins; HSF - heat shock factor proteins; Jumonji - proteins that contain JmjN and JmjC domains and may be protein hydroxylases that catalyse a novel histone modification; LBD and LOB-LATERAL ORGAN BOUNDARIES domain containing proteins which bind to GCGGCG; LSD - proteins that contain three zinc finger domains; M-type - similar to "MADS"; MADS - *MINICHROMOSOME MAINTENANCE 1 *(*MCM1*) from *S. cerevisiae AGAMOUS *(*AG*) from *Arabidopsis thaliana*, *DEFICIENS *(*DEF*) from *Antirrhinum majus *and *SERUM RESPONSE FACTOR *(*SRF*) from *Homo sapiens*) family members; MBF1 - proteins similar to multiprotein bridging factor 1 and mediators of transcriptional activation by bridging between an activator and a TATA-box binding protein (TBP); MIKC - type-II MADS-box containing proteins; mTERF - proteins containing repetitions of a 30 amino acid module, the mTERF motif, containing leucine zipper-like heptads; MYB - proteins containing the MYB (from the oncogene of avian myeloblastosis virus) domain; MYB-related - proteins containing MYB-related domains; NAC - named for NAM, ATAF, and CUC family members; NF-YA - the A subunit of the NF-Y complex that recognizes CCAAT box motifs; NF-YB - the B subunit of the NF-Y complex that recognizes CCAAT box motifs; Nin-like - nodule inception-like proteins; OFP - ovate family proteins contain a conserved C-terminal domain; Orphans - transcription factors that don't belong to any of the other families -- from GrassTFDB; PHD - proteins containing a Plant Homeo Domain finger that resembles the metal binding RING domain (Cys3-His-Cys4) and FYVE domain; PLATZ - proteins similar to PLATZ1 (plant AT-rich sequence- and zinc-binding protein 1) zinc-dependent DNA-binding protein; Pseudo ARR-B - type-B phospho-accepting response regulator proteins; RAV - proteins containing both a B3 domain and a single AP2/ERF domain; RWP-RK - proteins containing a RWP-RK domain; SBP - proteins encoding a conserved protein domain of 76 amino acids in length (SBP-domain); SET - proteins containing a 130-residue SET domain (named after three Drosophilia genes involved in epigenetic processes, Su(var), E(z) and trithorax); Sigma70-like - proteins similar to sigma70; SNF2 - proteins with seven characteristic blocks comprising the helicase region; TALE - three-amino-acid-loop-extension class of homeoproteins contains the KNOTTED-like homeodomain (KNOX) and BEL1-like Homeodomain (BELL) members; TAZ - proteins containing a TAZ2 zinc finger; TCP - proteins containing the TCP (from teosinte branched1from maize, *CYCLOIDEA *from snapdragon, and the *PROLIFERATING CELL FACTORS 1 *and *2 *from rice) domain, a 59-amino acid basic helix-loop-helix motif; Tify - proteins containing a TIFY domain (named for the most conserved amino acids); Trihelix - proteins containing helix-loop-helix-loop-helix domains; TUB - proteins containing C-terminal tubby domains; Uncategorized - transcription factors that don't belong to any of the other families -- GrassTFDB and PlnTFDB; WOX - homeobox proteins containing a conserved DNA-binding homeodomain; WRKY - proteins containing the WRKYGQK sequence followed by a C2H2 or C2HC zinc finger motif; YABBY - similar to "C2C2-YABBY"; ZF-HD - zinc finger homeodomain proteins Click here for file

Additional file 4 All genes differentially expressed under ABA- or PEG-treatment Click here for file

Additional file 5 **Oligonucleotides used for qRT-PCR**. ^a^GeneIDs in bold have unknown protein function. ^b^qPCR results comparing treated vs. control samples; passed - qPCR results agreed with RNA-Seq data; failed - qPCR results did not agree with RNA-Seq data. Click here for file

Additional file 6 **Enrichment p-values for GO biological processes categories**. ^a^GO categories falling under Other do so because they are found as enriched for groups of DE genes which are not easily grouped according to differential expression in tissue or treatment. Click here for file

Additional file 7 **SorghumCyc pathway Z-score enrichment analysis for genes differentially expressed in treatment vs. control roots and shoots**. ¹Observed Counts = The number of DE genes found within a given pathway. ^2^Expected Counts = The number of DE genes expected within a given pathway based on the size of the pathway and DE gene list (see Methods). ^3^A Z-score ≥ 2 with a corresponding p-value ≤ 0.05 is indicative of an enriched pathway. Click here for file

Additional file 8 **Ratio of reactions containing differentially expressed genes to the total number of reactions within annotated pathways**. ^a^Pathways are only included in this list if they contain 3 or more reactions. Click here for file

Additional file 9 **p-Values for *cis*-element enrichment within 1000 bp from the transcription start sites for differentially expressed genes in response to ABA- and PEG-treatment**. †B (CGT); D (AGT); H (ACT); K (GT); M (AC); N (ACGT); R (AG); S (CG); V (ACG); W (AT); Y (CT) Click here for file

Additional file 10 **Ortholog gene pairs of unknown function important in drought response**. ^a^[145]; ^b^[26]; ^c^[13]; ^d^[17]; ^e^[30] Click here for file

Additional file 11 **p-Values for *cis*-element enrichment in 1000 bp upstream of the transcriptional start site for sorghum genes with unknown function conserved in function and expression across species**. †B (CGT); D (AGT); H (ACT); K (GT); M (AC); N (ACGT); R (AG); S (CG); V (ACG); W (AT); Y (CT) Click here for file

Additional file 12 **p-Values for *cis*-element enrichment in 1000 bp upstream of the transcriptional start site for rice, maize, and Arabidopsis genes with unknown function conserved in function and expression to sorghum**. †B (CGT); D (AGT); H (ACT); K (GT); M (AC); N (ACGT); R (AG); S (CG); V (ACG); W (AT); Y (CT) Click here for file

Additional file 13 Number of genes within read count bins Click here for file
